# Comparison of right and left trans-radial catheterization for coronary angiography and intervention in patients with Acute myocardial infarction

**DOI:** 10.12669/pjms.333.12562

**Published:** 2017

**Authors:** Hongying Wang, Shijin Song

**Affiliations:** 1 Hongying Wang, Department of Cardiology, Tianjin 4th Centre Hospital, Tianjin-300140, China; 2 Shijin Song, Department of Cardiology, Tianjin 4th Centre Hospital, Tianjin-300140, China

**Keywords:** Acute myocardial infarction, Fluoroscopy time, Interventional procedural time, Left radial approach, Right radial approach

## Abstract

**Objective:**

To compare the clinical characteristics between the right and left radial approach in treating acute myocardial infarction, helping physicians make treatment strategies correctly.

**Methods:**

The patients admitted at our institution and undergoing percutaneous coronary angiography and interventional procedures by left or right radial approach between November 2013 and July 2016 were retrospectively reviewed. The access time, compression time, ambulation time, the amount of contrast material used, fluoroscopy time, interventional procedural time, the number of catheters used, the percentage of procedures completed using the assigned approach and the major vascular complications were recorded and compared between the two groups.

**Results:**

There were no significant differences in access time, compression time, the amount of contrast material used, number of catheters used as well as the time to ambulation between the two groups (p>0.05), but the fluoroscopy time and interventional procedural time were significantly longer in right radial approach group than those in left radial approach group (p<0.05). The left radial approach group presented with a higher percentage of procedures completed using the assigned approach than that of right radial approach group (p<0.05).

**Conclusion:**

The left radial approach has more advantages than right radial approach in treating acute myocardial infarction.

## INTRODUCTION

Cardiovascular disease is the leading cause of mortality, which causes approximately 13% of all deaths in the world.^1^ Acute myocardial infarction is the most serious presentation of coronary heart disease and its treatment has been paid high attention to,^2^ and the short-term treatment goal for it is to restore blood flow in the infarct related artery (IRA).^3^ Over the past 20 years, there has been considerable progress with improved outcomes in treating acute myocardial infarction.

Currently, percutaneous coronary intervention is widely used and well accepted in the treatment of this fatal disease.^4^ Percutaneous coronary intervention can be carried out via transfemoral or transradial approach. Some authors reported that the transradial approach was as safe and efficient as the transfemoral approach.^5^ In addition, the recent literatures demonstrated, compared to transfemoral approach, transradial approach presented with better clinical outcomes and shorter duration of hospitalization, because it can reduce vascular complications and bleeding of percutaneous coronary interventonal procedures.^5^,^6^

However, which is better, left radial or right radial approach? Some studies have been performed to compare the left and right radial approaches. In a study from Kanei, one hundred ninety-three patients were randomized to the right or left radial approach, The clinical outcome revealed that the procedural difficulty, fluoroscopy time, and contrast use were similar between the two groups, but the use of a single catheter was more common in the right radial group.^7^ In Freixa’s study on one hundred octogenarian patients, the procedural and fluoroscopy times were also similar in the two study groups.^8^ While, in one study of 1467 patients, Sciahbasi found that left radial approach was associated with lower fluoroscopy time and radiation dose adsorbed by patients compared with the right approach.^9^ These studies lead to different conclusions, and there were controversial viewpoints in terms of the procedural difficulty, fluoroscopy time, and contrast use between left and right radial approach.

Therefore, in the present study we reviewed retrospectively the patients with acute myocardial infarction treated using transradial approach. The aim of this study was to compare the clinical characteristics between the right and left radial approach, and help physicians make treatment strategies correctly.

## METHODS

The patients admitted at our institution and undergoing percutaneous coronary angiography and interventional procedures by either transradial approach between November 2013 and July 2016 were retrospectively reviewed. The demographic, clinical, and procedural data were collected. The study was approved by the ethics committee of our hospital. The Allen test was carried out in all the patients assigned to the transradial intervention, and abnormal test was contraindication to transradial catheterization. In addition, patients with acute coronary syndromes and with history of coronary artery bypass graft surgery were excluded.

The operator performed all the procedures standing on the right side of the patient for both left and right radial approaches. After local subcutaneous anesthesia, the radial artery was punctured with a 20 gauge needle. A 0.048 guidewire was introduced through the needle, followed by the insertion of a 5F or 6 F sheath, and then 2,500 IU of heparin was administered through the sheath. In case of radial spasm, direct arterial injection of nitrates or verapamil was carried out.^9^ After completion of the coronary angiography and intervention, the catheter was withdrawn and the arterial sheath was removed and hemostasis achieved by compression and pressure bandage.

In this study, the access time, compression time, ambulation time, the amount of contrast material used, fluoroscopy time, interventional procedural time, and the number of catheters used were recorded and compared between the two groups. The access time was defined as the time from the initiation of local anesthesia to completion of the insertion of the sheath, the interventional procedure time was the time from the introduction of the guide wire/catheter to the completion of angioplasty,^10^ and the compression time was the time necessary to achieve adequate hemostasis at the access site.^10^ Time to ambulation was defined as the time it took for the patient to sit up and ambulate after the procedure. In addition, the percentage of procedures completed using the assigned approach and the major vascular complications were recorded in the included patients.

### Statistical Analysis

Statistical analysis was performed using SPSS 21.0 (SPSS Inc., Chicago, IL, USA). The continuous variables were evaluated by independent 2-sample t-test, and categorical variables by Chi-square test or Fisher’s exact test between the two groups. P<0.05 was considered to indicate statistical significance.

## RESULTS

In the current study, 889 patients were included. Of the 889 patients, 551 patients underwent a diagnostic coronary angiography (298 patients were assigned in left radial approach group and 253 patients in right radial approach group), and in 338 patients, a percutaneous coronary intervention was carried out after the diagnostic angiography (181 patients were assigned in left radial approach group and 157 patients in right radial approach group). There were no significant differences in baseline characteristics including age, gender, height, weight, smoking history, surgical history and chronic medical conditions between the left and right radial approach group (p>0.05).

**Fig.1:**
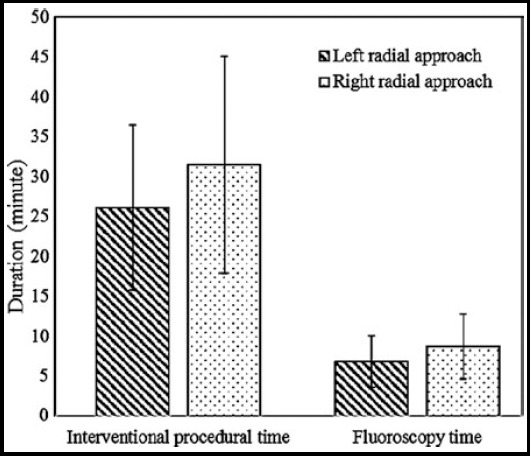
The interventional procedure time and fluoroscopy time of the two groups.

In terms of the characteristics of the procedures, there were no significant differences in access time, compression time, contrast medium administered, number of catheters used as well as the time to ambulation between the two groups (p>0.05, Table-I) but the fluoroscopy time and interventional procedural time were significantly longer in right radial approach group than those in left radial approach group (p<0.05, Fig.1). In 479 patients assigned into left radial approach group, procedures were completed successfully in 470 cases and the percentage was 98.1%, and in 410 patients assigned into right radial approach group, procedures were completed successfully in 391 patients and the percentage was 95.4%. There was significant difference in the percentage of procedures completed using the assigned approach between the two groups (p<0.05).

**Table-I:** The characteristics of the procedures in the two groups.

	*Left radial approach*	*Right radial approach*	*P value*
Access time (min)	1.8±0.5	2.1±0.7	P=0.67
Compression time (min)	7.3±1.8	7.5±1.7	P=0.23
Fluoroscopy time (min)	6.8±3.2	8.7±4.1	P=0.04
Interventional procedural time (min)	26.1±10.3	31.5±13.6	P=0.03
Contrast use, CA (mL)	109±54	112±59	P=0.78
Contrast use, PCI (mL)	158±87	162±79	P=0.92
Number of catheters used			P=0.86
Two	398	340	
Three	66	56	
Four	15	14	
Time to ambulation (hour)	10.1±4.6	11.7±5.3	P=0.35

The procedure could not be completed in 28 patients mainly because of tortuosity or calcification of the subclavian artery in 19 patients (14 in right radial approach and five in left radial approach group, p=0.015) and for reasons related to the radial artery in nine patients (four in right radial approach and 5 in left radial approach group, p=0.92). All the 28 cases were shifted to transfemoral approach.

In terms of the vascular complications, stroke occurred in one patient in the right radial approach group, but none in the left radial approach group (P = 0.28). Access-site hematomas was found in three cases of right radial approach group and two cases of left radial approach group (p=0.53). There were no cases of pseudoaneurysm, arteriovenous fistula, or ischemic complication of the arm in the study patients.

## DISCUSSION

In the current study, we found the left radial approach was associated with shorter fluoroscopy time and procedure time compared with the right radial approach. Earlier studies have also shown similar results. Sciahbasi found in his study that thefluoroscopy time was shorter in the left than in the right approach group.^9^ In a study of 437 patients, Kawashima also found both the duration of catheter manipulation and the total procedure were shorter in the left than in the right approach group.^11^ These results may have been contributed to by two reasons. On one hand, a series of anatomical variations are available during the arterial path from both wrists to the ascending aorta; at another hand, the left radial approach may be related to a lower impact of subclavian tortuosity, which is a major issue in prolonging the length of the procedure because of increased difficulty in catheter manipulation.^9^

In addition, the left radial approach group presented with a higher percentage of procedures completed using the assigned approach than that of right radial approach. Similarly, the impact of subclavian tortuosity may lead to difficulty of access when the right radial approach is carried out, which may affect the success rate of right radial approach adversely. In a study of 1005 consecutive patients carried out by Santas,^10^ 71% procedures were completed successfully with left radial approach and 68% with right radial approach. In comparison to Santas’ study, the percentage of procedures completed using the left or right radial approach in the current study was far higher. The approaches were assigned randomly, and contraindications were not considered in Santas’ study, which resulted in a relatively lower percentage of procedures completed using left or right radial approaches.

At the same time, among the 19 patients who failed in the radial approach and were shifted to transfemoral approach because of tortuosity or calcification of the subclavian artery, 14 were from right radial approach group and 5 in left radial approach group, which further confirmed the above mentioned viewpoints. Simultaneously, we found in the current study there were no significant differences in the occurrence of the complications such as stroke and access-site hematomas between the two groups. This together with the shorter fluoroscopy time and procedure time demonstrate the advantages of left radial approach.

### Limitations of the study.

First, the study was carried out retrospectively, while a prospective randomized controlled study may be better in clarifying the issues. Second, the sample size was relatively small in the current study; a larger scale study may demonstrate the facts more significantly. However, despite these limitations, we can conclude from the present study that the left radial approach has more advantages than right radial approach in treating acute myocardial infarction; this may facilitate physicians in determining the diagnosis and treatment strategies of the fatal disease.

### Authors’ Contribution

**SJS** conceived, designed and did statistical analysis & editing of manuscript.

**HYW, SJS** did data collection and manuscript writing, and final approval of manuscript.
